# The Biochemical Landscape of Riboswitch Ligands

**DOI:** 10.1021/acs.biochem.1c00765

**Published:** 2022-01-24

**Authors:** Ronald R. Breaker

**Affiliations:** Department of Molecular, Cellular and Developmental Biology, Yale University, New Haven, CT 06520-8103, USA;; Howard Hughes Medical Institute, Yale University, New Haven, CT 06520-8103, USA;; Department of Molecular Biophysics and Biochemistry, Yale University, New Haven, CT 06520-8103, USA

**Keywords:** aptamer, bacterial gene control, coenzyme, noncoding RNA, RNA World

## Abstract

Over 55 distinct classes of riboswitches that respond to small metabolites or elemental ions have been experimentally validated to date. The ligands sensed by these riboswitches are biased in favor of fundamental compounds or ions that are likely to have been relevant to ancient forms of life, including those that might have populated the “RNA World”, which is a proposed biochemical era that predates the evolutionary emergence of DNA and proteins. In the following text, I discuss the various types of ligands sensed by some of the most common riboswitches present in modern bacterial cells and consider implications for ancient biological processes based on the proven capabilities of these RNA-based sensors. Although most major biochemical aspects of metabolism are represented by known riboswitch classes, there are striking sensory gaps in some key areas. These gaps could reveal weaknesses in the performance capabilities of RNA that could have hampered RNA World evolution, or these could highlight opportunities to discover additional riboswitch classes that sense essential metabolites.

## Graphical Abstract

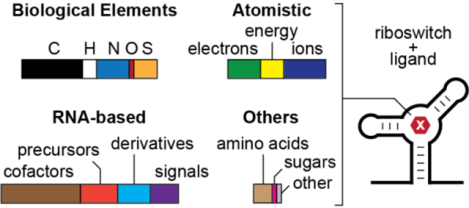

In the last two decades, the collection of experimentally validated riboswitch^1–5^ classes has been steadily growing^6^. These RNA-based gene-control devices (Figure 1) sense small molecules or elemental ions and undergo structural changes to modulate the expression of relevant genes. Bacterial riboswitches are commonly located in the 5′-untranslated regions of certain messenger RNAs, where each carry at least one ‘aptamer’ domain that selectively binds its target ligand^7–9^. The aptamer structure also interfaces with an overlapping ‘expression platform’ to modulate one of several different processes that affects protein production (Figure 1A). For the present analysis, riboswitch representatives are organized into distinct classes either if they use an aptamer with a unique folded architecture or if they use a familiar fold but sense a different ligand (Figure 1B). Using these criteria, there are at least 56 riboswitch classes reported to date that have strong support for their biochemical and/or genetic function (Figure 2).

It has been proposed that the known riboswitch collection represents only a tiny fraction of the classes that likely exist amongst the global bacterial population^6,10,11^. Support for this view is derived from the fact that there appear to be exponentially more classes as the abundances of each class decreases, such that the distribution follows a power law equation^12^. This trend is partly observable by examining the numbers of representatives for the known riboswitch classes presented herein (Figure 2, ‘Count’ column). However, this list is imperfect because not all riboswitch classes, with for example 10 or more representatives, have been discovered. This incomplete dataset causes uncertainty in the projections made by applying a power law analysis, which makes such estimates inherently imprecise. Regardless, it seems much more probable to encounter an abundantly represented riboswitch class via genetic or bioinformatic search methods than an exceedingly rare class. If true, then future riboswitch discoveries might only occasionally place a novel class among the top 25 most abundant riboswitches even though many thousands of riboswitch classes remain to be discovered.

If the approximations noted above are reasonable, then the current collection of riboswitch classes reflect the functions of some of the most abundant riboswitch classes in modern bacterial species. Even the rare classes on the present list (Figure 2) might represent relatively abundant classes as the list is expanded by future riboswitch discoveries. By considering the biological roles of the ligands sensed by these RNAs, it is possible to determine what types of biochemical processes are overseen by riboswitches that are used by cells despite the expected strong evolutionary competition from modern protein genetic factors. Even classes that prove to be exceedingly rare, at a minimum, provide insights into the capabilities of RNA-based receptors and genetic switches to form selective binding sites for biologically relevant ligands, and to modulate the action of biochemical processes. Herein, I group the known riboswitch ligands into various key categories and discuss the implications for riboswitch function in modern and ancient forms of life.

## RNA WORLD ORIGIN FOR RIBOSWITCHES

Numerous reasons have been given for the likely emergence of riboswitches during the RNA World^6,11,13–15^, which is a proposed era in evolution where functional RNAs predated proteins^16,17^. In part, this hypothesis is supported by the observation that many riboswitch classes in modern cells sense ligands that are building blocks or derivatives of RNA nucleotides or their precursors^6,11,15^. Particularly notable is the fact that more than half of the common enzyme cofactors and RNA-based signaling molecules have at least one corresponding riboswitch class (Figure 3). This is relevant because most of the enzyme cofactors either carry ribonucleotide moieties or are derived from ribonucleotides or their precursors, which have been proposed to reflect their ancient RNA World origin^18,19^. Furthermore, coenzymes perform essential roles in many fundamental metabolic processes present in all domains of life. The fact that known riboswitches selectively bind many coenzymes indicates that these modern RNA aptamers might reflect structures and functions present in ancient forms of life. Perhaps some riboswitch structures are direct evolutionary descendants of ribozymes that synthesized enzyme cofactors or that used them to promote early metabolic processes^11,15^.

**Figure 2.**
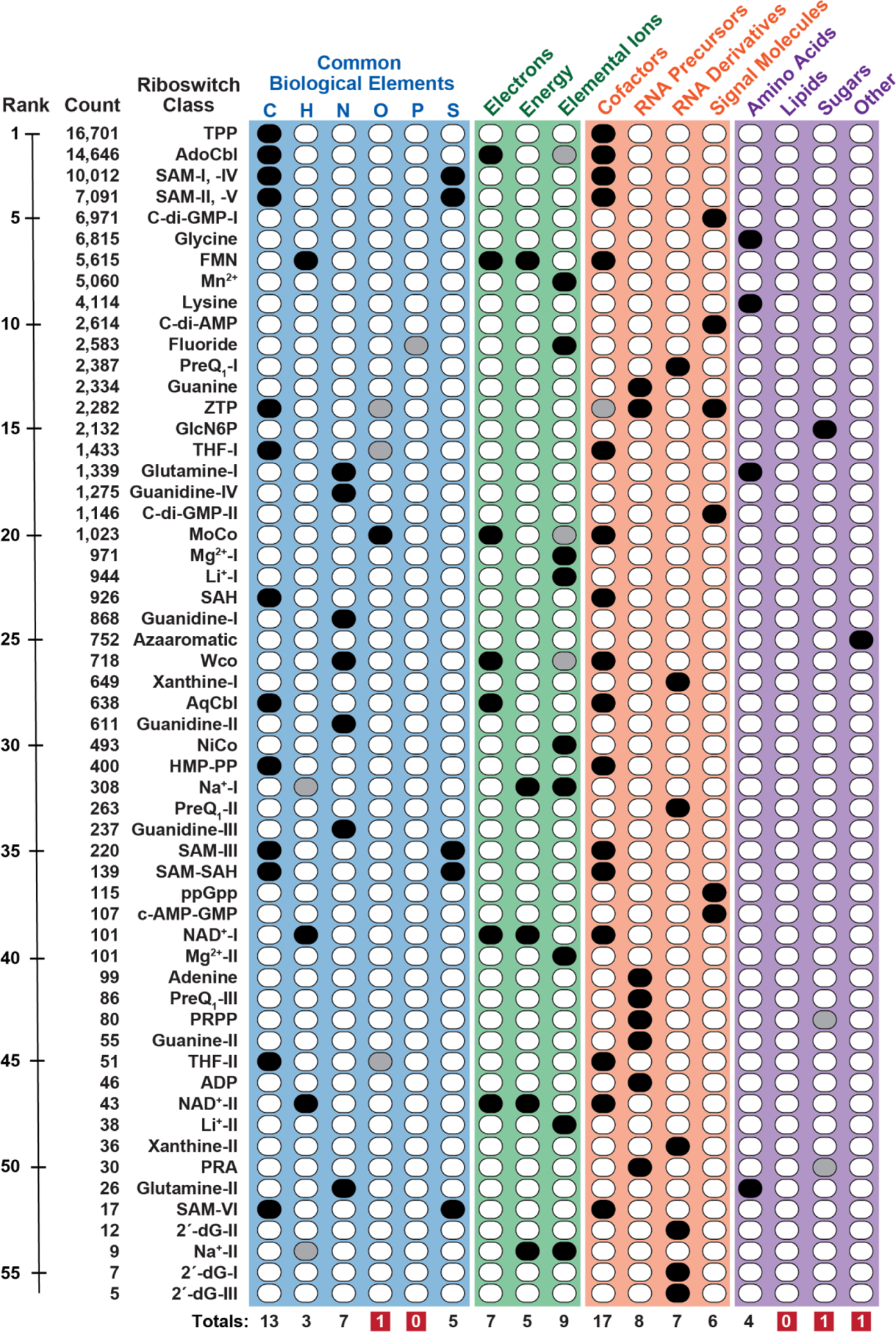
The list of riboswitch classes with strong experimental support validating their function. Each riboswitch class is named for the ligand sensed, where roman numerals designate distinct riboswitch classes for the same ligand. Black and gray ovals, respectively, indicate that the riboswitch ligand is strongly or weakly associated with the category named for each column. Biochemical categories evaluated include the common biological elements (blue), atomistic components: electrons, energy or elemental ions (green), RNA-based molecules (orange), or various other compounds (purple). Values below each column indicate how many riboswitch classes are known to sense ligands whose primary role (black oval) is linked to the named category. Values highlighted in red appear underrepresented with riboswitch classes. *Notes:* (*i*) Some riboswitch classes (SAM-I and SAM-IV; SAM-II and SAM-V) are listed on one line because their architectures are highly similar. (*ii*) Some riboswitch classes are designated as relevant to more than one category. (*iii*) It seems likely that many group I self-splicing ribozymes function as riboswitches for guanosine or any of its 5′ phosphorylated derivatives^15,100^, however these are not included as a riboswitch class on the current list due to ambiguity with their function as selfish genetic elements. (*iv*) Several additional proposed riboswitch classes have been reported in the literature, but these are excluded from the list due to insufficient experimental evidence.

Even if today’s riboswitch sequences were not directly derived from ribozymes or aptamers present in the RNA World, they at least reveal the types of functions that were possible if only RNA polymers are used to construct ligand receptors. For example, six major RNA architectures exist that selectively sense the coenzyme *S*-adenosylmethionine (SAM) or its metabolic breakdown product *S*-adenosylhomocysteine (SAH) (Figure 2)^6,20,21^. Four different riboswitch classes exist for the nitrogen-rich and toxic guanidinium ion^22–26^. These findings reveal that the structural potential of RNA is sufficient to form numerous functionally equivalent aptamers for some ligands.

Just as riboswitch aptamers for some target ligands are structurally diverse, there is also considerable proven diversity in the types of ligands sensed by natural riboswitches (Figure 2). As discussed above, enzyme cofactors are the best represented category for riboswitch ligands. However, numerous other ligand types are recognized by distinct riboswitch classes, which means that ancient RNA World organisms could have exploited RNAs to monitor large sectors of biochemical processes essential for their survival. Some of the biochemical categories represented by known riboswitch ligands are discussed in greater detail below.

## MANAGING ‘CHNOPS’ ATOMS

All cells must coordinate the uptake and utilization of the major atoms used in biological processes, namely carbon, hydrogen, nitrogen, oxygen, phosphorus, and sulfur (CHNOPS). These elements comprise the vast majority of atoms forming nucleic acids, proteins, sugars, and lipids that are essential for all forms of life. To thrive, modern cells (as would have their evolutionarily advanced RNA World ancestors) need to monitor the levels of these atoms and adjust their cellular concentrations as necessary. An analysis of known riboswitch ligands indicates that bacterial riboswitches demonstrate a broad capability for monitoring most CHNOPS elements. However, there are some notable gaps that are also discussed below.

### Carbon Management by Riboswitches is Robust.

Among the known riboswitch classes are numerous aptamers that sense coenzymes involved in manipulating carbon atoms (Figure 2). Indeed, the four most abundant riboswitch classes sense thiamin pyrophosphate (TPP)^27^, adenosylcobalamin (AdoCbl)^13^, or SAM (two classes)^20^, which carry out chemical transformations involving carbon centers such as decarboxylation and methyl transfer reactions. This list is augmented by a riboswitch class that detects the TPP biosynthetic precursor 4-amino-2-methyl-5-hydroxymethylpyrimidine pyrophosphate (HMP-PP)^28^, an AdoCbl riboswitch variant that senses aquocobalamin (AqCbl)^29,30^, and four additional rarer riboswitch classes that sense SAM or its metabolic waste product SAH^20,21^.

Furthermore, the remarkably versatile cofactor tetrahydrofolate (THF), which is sensed by at least two riboswitch classes^31,32^, promotes chemical reactions with single carbon units at different levels of oxidation^33^. Surveillance of single carbon units is further achieved in some species via the use of ZTP riboswitches^34^, which sense phosphorylated derivatives of the purine biosynthetic precursor 5-Aminoimidazole-4-carboxamide-1-beta-D-ribofuranosyl 5’-monophosphate (AICAR). A shortage of 10-formyl-THF (10f-THF) leads to the accumulation of AICAR, which can be further phosphorylated to form the bacterial alarmone called ZTP^35^. Members of the ZTP riboswitch class subsequently activate the expression of genes involved in 10f-THF biosynthesis^34^. Thus, ZTP directly participates in carbon management by monitoring for an adverse metabolic effect of either THF or carbon unit depletion.

**Figure 3.**
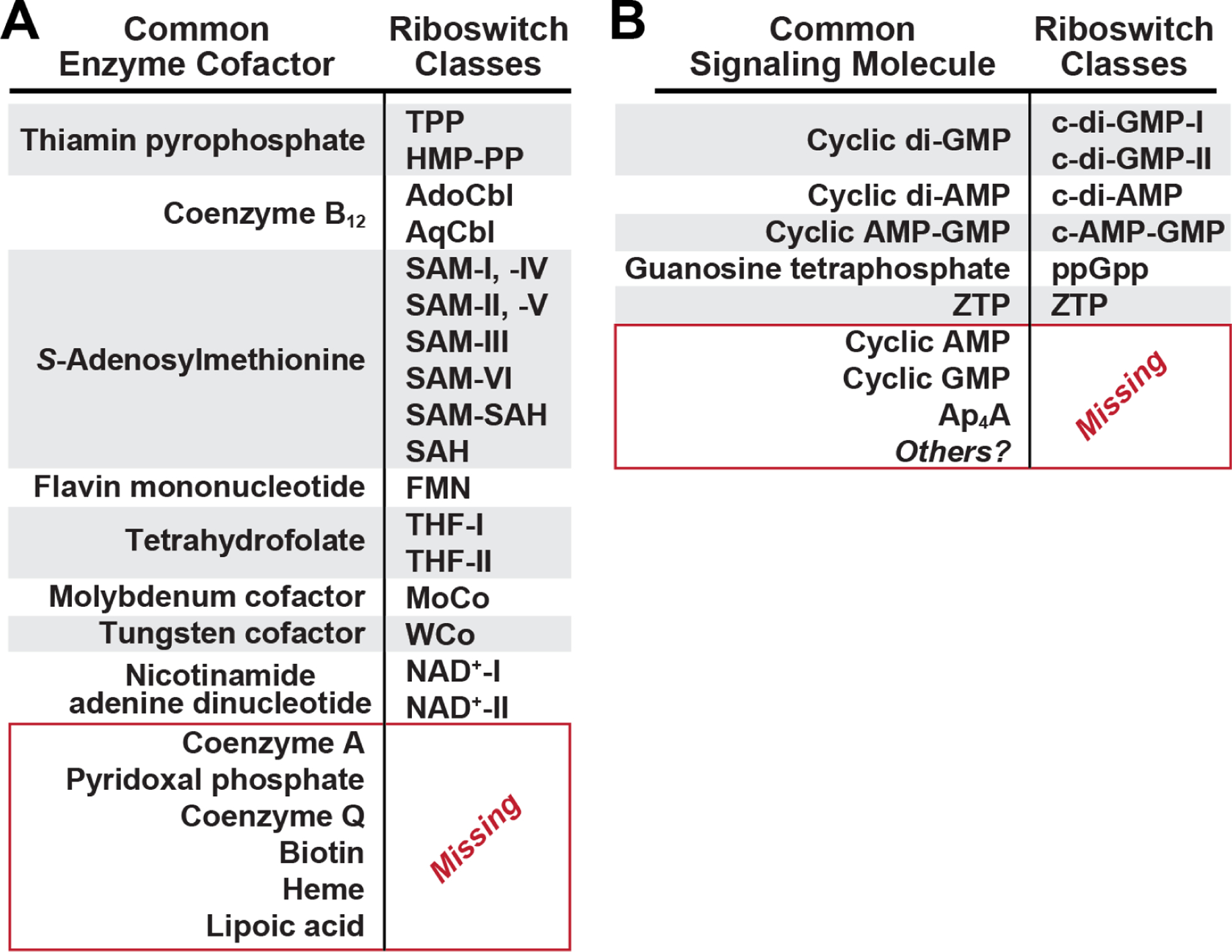
Many riboswitch classes have been discovered for the most widely distributed enzyme cofactors (A) and nucleotide-based signaling molecules (B).

In total, 13 of the known riboswitch classes are involved in sensing ligands relevant to carbon management (Figure 2). Twelve of these classes sense enzyme cofactors that are directly involved in manipulating single carbon units. This fits with the striking theme noted above that many cofactors are sensed by riboswitches^6,11,36^. However, there are a few types of enzyme cofactors that are surprisingly lacking a known riboswitch sensor (Figure 3A). Among these are coenzyme A (CoA)^37^, which serves as a carrier of different-length alkyl chains via a thioester linkage, and biotin^38^, which is commonly involved in carboxylation reactions. The roles of CoA for management of carbon polymers (such as fatty acids) and the roles of biotin in the process of integrating (fixing) the carbon from CO_2_ into biological molecules are of fundamental importance. Thus, the absence of riboswitches for these two cofactors leaves a notable blind spot for RNA-based sensors, which could have been problematic for RNA World organisms that could not exploit protein factors to carry out these sensory tasks.

Although, it seems likely that selective CoA-responsive riboswitches were present in RNA World organisms, evidence supporting this hypothesis is notably lacking. Indeed, engineered RNAs have been made that recognize CoA or its derivatives^39–41^, but unfortunately these RNAs primarily recognize the adenosyl moiety of the coenzyme and thus cannot be used to prove that RNA is capable of selective CoA recognition. Even if CoA riboswitches can be formed, perhaps they are rare in modern cells and thus are difficult to discover, or those classes present in the RNA World have since been lost through evolutionary competition with protein factors.

### Hydrogen Management with Hydride Carriers.

Protons are rarely in short supply in biological systems because water (at ~55.5 M when pure) serves as the solvent for living systems. Even under extreme alkaline conditions, a proton can be obtained from a water molecule in a short period of time, although some enzyme active sites exclude water to avoid unwanted reactions. There seems little need for the cell to closely monitor hydrogen amounts as it relates to supplying building materials for biological compounds.

However, there are other concerns relevant to hydrogen that cells must address, such as managing concentration of free protons and the chemical handling of hydride (H^−^) units. Forming RNAs that precisely monitor the pH of cells seems like an easy task given the p*K*a values of some nucleobases^42^. However, there currently are no known riboswitches that serve as proton sensors, either by directly binding to H^+^ or by indirectly sensing distress caused by extreme pH conditions. The closest any known riboswitch class comes to proton management is found with Na^+^-I riboswitches^43^. These riboswitches sense Na^+^ as the name indicates and frequently regulate Na^+^/H^+^ antiporters that operate best under alkaline conditions. This permits cells to exploit an Na^+^ gradient to import H^+^ when cells are living in alkaline environments, thereby maintaining the desired near-neutral pH inside cells.

An abundant riboswitch class exists for flavin mononucleotide (FMN)^44,45^ and two rare classes have been discovered that sense nicotinamide adenine dinucleotide (NAD^+^)^46,47^ (Figure 2). These ligands incorporate either a flavin^48^ or a nicotinamide^49^ chemical moiety that serve as the predominant carriers of hydride units in all forms of life. Although these cofactors or their close derivatives cover a large portion of hydride carrying tasks in modern cells, it is notable that riboswitches could further expand their role in hydride ion management if they were to sense the near ubiquitous coenzyme Q (CoQ)^50^, which currently lacks a corresponding riboswitch (Figure 3A). CoQ, either as menaquinone or plastoquinone, commonly exists in a form linked to a long alkyl chain whose hydrophobic nature favors its association with cell membranes. Perhaps this hydrophobic character restricts the ability of RNA (a polyanion) to form a selective binding pocket for CoQ, although presumably precursors of this coenzyme end-product that are less hydrophobic and available in the cytosol could be more readily bound by an RNA aptamer.

### Nitrogen Management is Well Represented by Riboswitch Ligands.

The distribution of nitrogen into amino acids is commonly achieved by transamination reactions where the source of amino groups is glutamate (the most abundant organic compound in cells)^51^. The concentration of the similar amino acid glutamine, which carries an additional nitrogen atom compared to glutamate, is used by many bacteria as a measure of nitrogen abundance^52^. Two riboswitch classes, called glutamine-I^53^ and glutamine-II^54^, form structures that are nearly superimposable^55,56^ and are associated with genes relevant to nitrogen management in various bacterial species. For example, species of cyanobacteria use glutamine-II riboswitches to regulate the expression of glutamine synthase, which is the key enzyme for nitrogen assimilation in many organisms^54^. Thus, riboswitches directly participate in the regulation of nitrogen levels in cells.

Intriguingly, there are four different classes of riboswitches that sense the cation form of the nitrogen-rich compound guanidine (Figure 2)^22–26^. Guanidine, which carries three nitrogen atoms, appears to accumulate to toxic levels unless a riboswitch activates the expression of genes whose protein products either eject the molecule or promote its breakdown into CO_2_ and ammonium ions^22^. In contrast, it has been proposed^57^ that at least some guanidine transporters import the compound for utilization as a nitrogen source. In such cases, guanidine riboswitches might function to suppress expression of the transporters so that guanidine does not accumulate to toxic levels. Unfortunately, the natural sources of guanidine have yet to be identified, but guanidine-sensing riboswitches appear to play an important role in adjusting nitrogen levels in cells when it accumulates in the form of guanidine^57–60^.

### Oxygen Management is Poorly Represented by Riboswitch Ligands.

Ligands relevant to oxygen management are notably scarce. Only molybdenum cofactor (MoCo)^61^ is counted as a riboswitch ligand whose primary function is relevant to this important task. The predominant role for MoCo-dependent enzymes is to promote oxygen transfer reactions involving redox processes^62,63^. Perhaps there is little need for organisms to monitor ligands relevant to oxygen because of its striking abundance. Again, water provides an ample source of hydroxyl groups that can supply oxyanion nucleophiles for hydrolysis or hydroxylation reactions. Similarly, the removal of oxygen during dehydration reactions produces a water molecule that simply adds to the surrounding solvent.

The scarcity of riboswitches for ligands relevant to oxygen management is somewhat addressed by riboswitches for THF and for the alarmone ZTP. Although most natural folate derivatives are considered members of the single carbon unit management system, one derivative, 10-formyl-THF (10f-THF), functions as a carrier of a carbon atom that is almost fully oxidized. Transfer of the formyl group from 10f-THF to a substrate thus constitutes the transfer of both carbon and oxygen atoms. In many bacteria, an adequate supply of 10f-THF is maintained by the actions of THF-I^31^ or THF-II^32^ riboswitches. Furthermore, as described above, a shortage of this enzyme cofactor also leads to the accumulation of ZTP^35^, which is sensed by a riboswitch class that activates the expression of genes involved in 10f-THF biosynthesis^34^. Thus, riboswitches for THF and ZTP also indirectly participate in oxygen management.

There are also currently no known riboswitch ligands that are relevant to diatomic oxygen, which is the terminal electron receptor for electrons in aerobic organisms that derive energy from hydride carriers (e.g. from NADH and FADH_2_) via oxidative phosphorylation^64^. Extant bacterial species also are known to use extensive protein-based systems to detect and mitigate the effects of reactive oxygen species (ROS)^65^. However, there is only a single rare riboswitch candidate class that selectively binds 8-oxoguanine, which might allow cells to detect the oxidative damage caused by ROS^66^. Perhaps riboswitches related to diatomic oxygen metabolism, including those for reactive oxygen species, are scarce because RNA World organisms might have thrived in an era without atmospheric molecular oxygen^67,68^, and therefore riboswitch relics for ligands relevant to these molecules would not be present in modern organisms. Regardless, additional riboswitch classes might remain to be discovered that manage metabolic processes related to oxygen management.

### No Evidence of Direct Phosphorus Management by Riboswitches.

The most remarkable observation among the existing riboswitch classes list is that there is not a single ligand that is directly related to phosphorus or phosphate homeostasis. To be clear, 22 of the 56 riboswitch classes listed herein (Figure 2) bind to ligands that carry one or more phosphate groups, and many of the aptamers of these classes form ligand binding pockets that exploit molecular recognition contacts with phosphates. Therefore, RNA molecules can readily form binding pockets that can recognize the major biochemical form for phosphorus.

The 22 riboswitch classes noted above sense 18 different ligands that carry at least one phosphate group. However, none of these ligands have any direct role that is relevant to phosphorus homeostasis. In contrast, many bacterial species manage phosphorus levels using a protein system encoded by the Pho regulon^69^. This system includes enzymes secreted to scavenge for phosphates, phosphate transporters, enzymes involved in storing phosphate, and regulatory proteins. So far, no riboswitches have been identified that exhibit extensive evidence of the regulation of genes relevant to the Pho system.

Two riboswitch classes do modulate genes that can affect cellular phosphate levels, although their major roles are categorized differently. Na^+^-I riboswitches^43^ occasionally regulate the expression of genes for ATP synthase complexes powered by a Na^+^ gradient. The action of ATP synthase is to couple inorganic phosphate to ADP to generate ATP. Although this can change the concentration of free inorganic phosphate in the cell, it does not change the overall phosphorus content. Rather, the Na^+^-I riboswitch class seems more relevant to energy metabolism and elemental ion homeostasis rather than for phosphorus management.

Also, fluoride riboswitches^70^ occasionally are associated with genes coding for phosphate transporters or polyphosphate phosphatase. Fluoride can inhibit phosphatase enzymes and form complexes with phosphate (e.g. fluorapatite). Therefore, the genetic associations between fluoride riboswitches and phosphate homeostasis genes appear to be purely defensive strategies to mitigate the toxic effects of the fluoride ligand. Currently, it seems that cells are remarkably devoid of riboswitches that manage phosphorus levels. This probably was not the case during the RNA World, where phosphorus monitoring and homeostasis systems would have been just as important as it is in modern cells.

### Sulfur Management is Largely Achieved by SAM Riboswitches.

There are six distinct riboswitch classes that sense the enzyme cofactor SAM^6,20,21^ or its metabolic derivative SAH^71^. The latter class is exclusively associated with genes that recycle SAH to regenerate SAM. As expected, SAM riboswitches are commonly associated with genes coding for SAM synthase, which involves the coupling of methionine to a fragment of ATP to produce the final coenzyme structure^72^. In addition, SAM riboswitches are associated with numerous genes that code for enzymes involved in integrating sulfur into metabolites on the path to forming methionine and cysteine^20^. Thus, the concentration of SAM is commonly exploited by riboswitches to serve as a proxy for the levels of sulfur present throughout the disparate collection of metabolites that also carry this atom.

Despite the use of this essential coenzyme to read out the general pool of sulfur in cells, it seems likely that other riboswitch classes will be found that detect sulfur-containing molecules to assist in fine tuning the use of sulfur in some metabolic pathways. Although not a perfect example, HMP-PP riboswitches^28^ activate the expression of genes for the production of 4-methyl-5-β-hydroxyethylthiazole phosphate (HET-P) only when HMP-PP is abundant. The two compounds are then joined to form the complete coenzyme precursor thiamin phosphate. Thus, HMP-PP riboswitches commit the cell to making the sulfur-containing HET-P molecule only if they have sufficient HMP-PP to warrant the use of sulfur and the other resources needed to make the thiazole moiety found in the final TPP coenzyme.

## MANAGING ATOMISTIC COMPONENTS

Biological systems must also closely monitor fundamental processes that are manifest at the level of atoms or their constituents. For the present discussion, redox potential, energy production, and elemental ions have been grouped into this broad category (Figure 2). This grouping also reflects the fact that riboswitches are known that contribute to the regulation of more than one of these broad biological categories as described below.

### Riboswitches for High-energy Electron Carriers.

High energy electrons, derived either from glycolysis or from other oxidative metabolic processes, are commonly carried in the form of hydride units by the coenzymes that include nicotinamide or flavin moieties. These coenzymes thus serve a dual role as carriers of both protons and high-energy electrons, and also are relevant to both the redox potential of cells and the ability of cells to exploit the energy present in the hydride electrons they carry. As discussed above in the context of hydrogen management, the oxidized forms of the hydride carrying coenzymes FAD and NAD^+^ are sensed by known riboswitch classes^44–47^. The electrons in these molecules can be delivered to the electron transport chain to drive the formation of a proton or Na^+^ gradient that is eventually used to generate ATP^64^. By regulating FMN and NAD^+^ production, riboswitch classes for these coenzymes help ensure that cells have the molecules needed to carry high-energy electrons and to exploit these electrons to generate energy in the form of chemical gradients and phosphoanhydride linkages as found in ATP.

Additional riboswitch classes are known that sense other redox-active coenzymes. Specifically, riboswitches for AdoCbl^13^, AqCbl^29,30^, MoCo^61^, and its tungsten derivative WCo^61^, are known to participate in various important redox reactions. For example, MoCo^73^ and WCo^74^ are extensively used by organisms to carry out low redox-potential reactions, and thus broaden the scope of redox reactions that are biologically accessible. Conspicuously absent on the list of validated riboswitches classes are those for electron carriers based on the heme moiety (Figure 3A). Heme is a ubiquitous redox-active cofactor common to many electron transport chain proteins as well as other proteins that carry out diverse functions^75^. Perhaps such riboswitches exist but are rare due to the fact that the core heme structure is often derivatized to form various forms of the cofactor, thereby making it unlikely that one riboswitch aptamer would emerge to sense the heme pool. Also, many species initiate heme biosynthesis from glutamyl-tRNA^76^, which provides other possible means to regulate heme production without directly sensing this cofactor.

### Abundant Riboswitch Classes for Elemental Ions.

A growing list of elemental ions are known to be sensed by various riboswitch classes. The Na^+^-I riboswitch^43^ class is known to regulate Na^+^ transporters, which could allow cells to avoid accumulating toxic levels of this cation. As also noted earlier, some representatives of this riboswitch class regulate the expression of ATP synthase components that exploit a Na^+^ gradient to generate energy in the form phosphoanhydride linkages. In rare instances^43^, an Na^+^-I riboswitch is found in tandem with a riboswitch for the signaling molecule cyclic-di-adenosine phosphate (c-di-AMP)^77^. A general role for this signaling molecule is to regulate processes involved in overcoming osmotic stress^78^. This observation indicates that some cells monitor the concentration of an elemental ion (Na^+^) to make decisions regarding osmotic stress responses.

Several other riboswitch classes are known to directly sense the target ion and regulate genes for homeostasis or to avoid accumulating toxic levels of the ligand (Figure 2). Riboswitch classes for Na^+^, Mg^2+^, and Mn^2+^ directly bind these biologically useful ions and regulate genes accordingly^43,79–83^. Others for Li^+^, F^−^, and Ni^2+^ or Co^2+^ appear to sense toxic levels of the target ligand and regulate genes to reduce or mitigate the effects of the ion^70,79,84^. However, caution must be used when interpreting the ligand specificities of ion-responsive riboswitches because there is evidence that some of the riboswitch classes noted above might naturally sense other elemental ion ligands^85,86^, such as Fe^2+^.

Some elemental ions are indirectly sensed by riboswitches for small molecule ligands that integrate the ion into their structures. AdoCbl and AqCbl are formed using corrin rings that bind a cobalt cation via coordination with its four pyrrole nitrogen atoms. Representatives of these riboswitch classes occasionally are used to regulate genes associated with cobalt transport^87^. Similarly, riboswitches for MoCo and WCo are frequently associated with genes annotated as molybdate or tungstate transporters^61^. In these examples, the riboswitches regulate the uptake of the metals in their highest oxidation state, in their oxoanion forms rather than in their free cation forms. The analogous situation might exist for the sensing and transport of phosphorus. However, as noted above, a riboswitch class has yet to be found that commonly associates with phosphate transporters.

## RIBOSWITCHES AND RNA-BASED LIGANDS

The remarkable enrichment of RNA-derived compounds serving as riboswitch ligands has been extensively discussed previously^6,11,36,88^. This enrichment is highlighted on the list of riboswitch classes (Figure 2) in four categories: cofactors, RNA precursors, RNA derivatives, and signaling molecules. A total of 37 riboswitch classes from the 56 included on the list of currently validated classes bind ligands that are either directly derived from nucleotides or from their metabolic precursors. This strong bias in ligand composition is consistent with the hypothesis that many riboswitch classes emerged in evolution when RNA orchestrated a complex metabolic state^11,36^. Below are a few comments that add perspective to this general observation.

### Riboswitches for ancient cofactors.

The evidence of RNA World origins for enzyme cofactors is strong^16–19^. If true, then the predominant ancient coenzymes are probably largely identical to those found in species from all three domains of extant life. This means that ribozymes and riboswitches predating the emergence of genome-encoded proteins would have, respectively, exploited the same coenzymes to assist in biocatalysis and would have bound these compounds to regulate their production and utilization^11,36,88–90^. It appears that the coenzyme-utilizing ribozymes have not endured well in competition with their protein counterparts, as no examples of natural coenzyme-dependent ribozymes have been reported to date.

However, more than half of the near-ubiquitous enzyme cofactors are sensed by one or more riboswitch classes, which total 17 in number (Figure 3A). A robust representation of riboswitch classes for nucleotide-like coenzymes is precisely what should be expected if RNA aptamers from the RNA World persisted in evolution to give rise to modern riboswitches. Furthermore, given the fundamental processes promoted by coenzymes, this allows riboswitches to be involved in many of the ligand categories as already presented in the sections above.

### Riboswitches for RNA precursors and derivatives.

There is also a striking abundance of riboswitch classes for RNA nucleotide precursors or derivatives. Some of these ligands are not at all surprising to see on the list (Figure 2), given their fundamental roles in RNA biochemistry. These include the purines guanine^91^ and adenine^92^, as well as the ZTP alarmone generated when cells have insufficient 10f-THF to make purines^34,35^. Also on the list are ribose derivatives that are central to the production of purines, as evidence exists that phosphoribosyl pyrophosphate (PRPP)^93^ and phosphoribosylamine (PRA)^94^ are sensed by distinct riboswitch classes. Finally, the xanthine-I^95^ and -II^96^ riboswitch classes sense oxidized forms of purines enroute to disposal.

Interestingly, the most abundant riboswitch class for an RNA derivative binds the guanine analog called pre-queuosine_1_ (preQ_1_), which is ultimately joined to a ribose and altered at position 7 of the former purine ring to carry various chemical moieties upon incorporation into RNA^97,98^ or DNA^99^. This modified nucleobase is present in organisms from all three domains of life, suggesting that it might have also been used during the RNA World. At least three distinct riboswitch classes for preQ_1_ are present in modern bacteria^100–102^, which reflects the continued importance of this compound and its precursors. For example, members of the preQ_1_-I class^100^ bind preQ_0_ with similar affinity^103^. This compound is the immediate precursor to preQ_1_ but is also derivatized by some species to produce natural antibacterial compounds such as toyocamycin and sangivamycin^104^. Thus, some bacteria might use riboswitches to monitor the pool of preQ_0_ and preQ_1_ molecules to ensure a sufficient supply of these modified nucleobases.

Riboswitches for the eight common RNA and DNA nucleosides or their phosphorylated derivatives are notably rare. Three highly similar riboswitch classes have been experimentally validated for 2′-deoxyguanosine (2′-dG)^96,105,106^, but these are few in number and therefore are only narrowly distributed in bacteria. In addition, one riboswitch class has been found with preferred affinity for adenosine-5′-diphosphate (ADP)^107^. As expected, each of these riboswitches regulate genes that are involved in nucleotide metabolism.

Also worth noting in this section are group I self-splicing ribozymes^108^, which use guanosine, or any of its 5′ phosphorylated derivatives, as a substrate to initiate a two-stage phosphoester transfer reaction series to process precursor RNA transcripts^109^. It has been suggested that some group I ribozymes might naturally function as guanosine- (or GMP-, etc.) responsive riboswitches^15,110^. This hypothesis is supported by the observation that group I ribozymes commonly reside in genes whose products (including tRNAs and rRNAs) contribute to the process of translation^108^. It has long been known that GTP hydrolysis processes are integral to the function and regulation of ribosomes^111,112^, and thus it seems reasonable to imagine that guanosine-based compounds also are important for regulating the expression of RNA or protein components of the translation apparatus.

Notably absent from the list of riboswitch ligands is ATP, which is used by many enzymes as a source of energy to drive forward otherwise thermodynamically unfavorable processes. To be clear, bacterial RNA aptamers that tightly bind ATP have been reported as part (domain 1) of a riboswitch class now called NAD^+^-I^46,113^. However, it seems almost certain that the natural function of these riboswitches is to sense NAD^+^ and not ATP as has recently been proposed^114^. The genes associated with the NAD^+^-I riboswitch class are exclusively associated with NAD^+^ biosynthesis, and they are never seen associated with genes that might be expected to be regulated by ATP. Other claims of ATP riboswitches^115^ also have proven to be false^22,77,82,83,116^. Furthermore, group I ribozymes can be altered by a single mutation to accept an adenosine moiety as the substrate for the first step of ribozyme-mediated splicing^39,117,118^, but this change in substrate specificity is not observed in nature^119^. Thus, group I ribozymes could have been repurposed to function as ATP sensors, but this capability does not seem to have been broadly exploited by modern cells. Although the long-sought riboswitch for the universal energy currency of cells remains to be found, it is possible that there are no abundant RNAs that serve as ATP riboswitches in modern cells.

### Riboswitches for nucleotide-derived signaling molecules.

It has been proposed that many common nucleotide-based signaling molecules and second messengers form a type of chemical language used by RNA World organisms to address internal and external communication needs^120,121^. Consistent with this hypothesis is the fact that six abundant riboswitch classes have been reported that sense the nucleotide-derived signaling molecules c-di-GMP (two classes)^110,122^, c-di-AMP^77^, c-AMP-GMP^123,124^, ppGpp^125^, and ZTP^34^ (Figure 3B). Given the importance of these signaling molecules, additional classes are likely to be discovered in the future. Furthermore, there are several prominent nucleotide-based signaling molecules that currently lack a riboswitch sensor. For example, 3′,5′-cyclic adenosine monophosphate (cAMP)^126^ and diadenosine tetraphosphate (Ap_4_A)^127^ are two widespread signaling molecules that present ideal ligands for sensing by riboswitch classes yet to be discovered.

## FEW RIBOSWITCH CLASSES FOR ALL OTHER METABOLITES

Given the ligand categories used for this discussion so far, there are many metabolites common to cells that would fall into separate groups. Below are discussed the surprisingly few riboswitch classes that sense compounds that are somewhat removed biochemically from the main pathways relevant to RNA metabolism.

### Only Three Amino Acids are Directly Sensed by Riboswitches.

Bacterial cells invest tremendous resources to produce the 20 common amino acids and to polymerize them into functional polypeptides based on information stored in the genome. Yet only three amino acids currently have corresponding validated riboswitch classes, glycine^128^, lysine^129,130^, and glutamine^53,54^. Given the broad importance of regulating amino acid levels, it might seem puzzling why only these three amino acids are directly sensed by riboswitch aptamers. It seems reasonable to speculate that glutamine riboswitches were particularly valuable to ancient cells because they provided a means to maintain adequate levels of this amino acid while also monitoring nitrogen levels^54^. Perhaps riboswitches for glycine and lysine also served broader, fundamental roles in organisms from the RNA World and therefore the RNAs persist in some modern species because of this evolutionary legacy. Regardless, riboswitches in modern cells have stiff competition from other systems that sense deficits in amino acid concentrations.

A major competitor is the classic mechanism of transcription ‘attenuation’^131^, which involves the use of ribosomes to evaluate the availability of particular aminoacyl-tRNAs to dictate the expression of the associated main open reading frame (ORF). Insufficient amounts of an amino acid result in reduced levels of the corresponding aminoacyl-tRNA. This in turn causes slow translation through an upstream open reading frame (uORF) preceding the main ORF, which results because the uORF is enriched in codons for the tRNAs carrying the amino acid being monitored. Slow uORF translation commonly induces read-through of an intrinsic transcription terminator stem, whose structure is precluded from forming by an antiterminator stem due to reduced ribosome speed. As a result, full-length mRNA is produced and yields an increase in the translation of genes related to the synthesis of the amino acid or its corresponding aminoacyl-tRNA. In attenuation systems, the ligand sensor is not a riboswitch aptamer, but the entire ribosome apparatus, whose translational speed dictates the folding pathway of the nascent mRNA.

Another major competitor is the mechanism used by ‘T-box’ RNAs^132^ that sense specific uncharged tRNAs. These RNAs are similar to riboswitches, but where the tRNA ligand is sensed by the nascent mRNA partly via a region of Watson-Crick base-pairing^133,134^. Specifically, T-box RNAs use a “specifier sequence” to selectively recognize the anticodon sequence of the target tRNA. Although these RNAs do not directly bind amino acids, the uncharged tRNA ligand serves as a proxy for low amino acid concentrations. T-box RNAs have been identified for tRNAs corresponding to all 20 amino acids^135^. On binding uncharged tRNA, the T-box RNA most commonly activate genes related to tRNA charging, such as those coding for aminoacyl-tRNA synthases and proteins important for the synthesis or transport of amino acids.

For both attenuation and T-box mechanisms, it is relatively simple to change the amino acid selectivity because the major determinant of the sensor is the sequence of the codon (attenuation)^131^ or anticodon (T-box)^135^. This appears to be more advantageous from an evolutionary perspective because it is likely easier to adapt these systems to monitor different amino acids rather than evolve distinct riboswitch aptamers that directly bind each of the 20 amino acids. Thus, bacteria already make extensive use of RNA-based mechanisms for indirectly sensing amino acid concentrations that appear as though they emerged in evolution during a time when the RNA World was transitioning to the modern RNA-DNA-protein world we see in today’s organisms. This likely reduces the need for other gene control systems such as riboswitches, or RNA-binding protein factors such as HutP^136^ or TRAP^137^, that directly bind the amino acid.

### No Riboswitches for Ligands Related to Lipid Biochemistry.

So far, the list of validated riboswitch ligands is devoid of connections to lipids, fatty acids, or other hydrophobic, membrane-associated compounds. This highlights an obvious concern about the ability of RNA molecules, which are highly negatively charged polymers, to form binding pockets for predominantly hydrophobic molecules. However, it has been proposed that RNA World organisms might have used terpenoid compounds in place of hydrophobic molecules or amphipathic phospholipids as a building material for membrane formation^16^. Again, perhaps there was little need for RNA World species to evolve aptamers for the lipid molecules that are very common in extant species.

Two additional observations further highlight the noteworthy absence of riboswitch classes for hydrophobic molecules. First, one of the enzyme cofactors that currently lacks a validated riboswitch partner is CoQ^138^ (Figure 3A). In its membrane-associated form, the ubiquinone ring is attached to a repetitive terpenoid tail to form CoQ_10_. If both the enzyme cofactor moiety and the terpenoid tail are of ancient origin, then perhaps RNA aptamers once existed that could selectively bind the final coenzyme structure or its biosynthetic precursors.

Second, a major carrier of fatty acid chains is CoA, which uses a tethered sulfur atom attached to phosphorylated adenosine to (among other functions) form a thioester linkage to fatty acids of various chain lengths^37^. By coupling hydrophobic molecules to nucleotides, RNA World organisms could have improved their opportunities for forming selective binding pockets using RNA-based receptors. Thus, as noted above, it remains puzzling why no examples of CoA-binding riboswitches have been identified in modern species.

Despite the polyanionic and hydrophilic character of RNA molecules, RNA aptamers might be sufficiently capable of forming binding pockets with characteristics suitable for binding some hydrophobic molecules. An example of this characteristic is found with the ‘azaaromatic’ class of riboswitches^116^. The aptamers of this class are notable for their ability to bind a diverse collection of hydrophobic, planar molecules with remarkably high affinity. The RNAs associate with a gene that is otherwise frequently located near to PadR protein factors belonging to a class of receptors famous for their ability to sense a wide range of hydrophobic molecules^139,140^, suggesting that azaaromatic riboswitches and PadR-like protein factors are functionally equivalent^116^. These findings provide some optimism that riboswitches for other hydrophobic molecules could have once existed and might still be found in modern organisms.

### Unmodified Sugars Currently Lack Riboswitch Sensors.

Among the riboswitches listed herein (Figure 2), 22 classes sense ligands that carry at least one sugar moiety (usually ribose or deoxyribose). However, each sugar has been chemically modified such that the resulting molecule is no longer made exclusively of carbon, hydrogen, and oxygen atoms. Only one modified sugar, glucosamine-6-phosphate (GlcN6P) is sensed by a riboswitch class for the purpose of regulating a metabolic process directly involving sugar management. Representatives of this class, called the *glmS* ribozyme^141^, bind GlcN6P and undergo a self-cleaving reaction to break the mRNA, leading to rapid degradation of the ORF coding for the GlmS protein^142^. GlmS is a glutamine-fructose-6-phosphate aminotransferase enzyme that produces GlcN6P for use in cell wall construction.

The next-least modified sugar ligands sensed by riboswitch classes are PRPP^93^ and PRA^94^. However, these riboswitches are associated with genes for nucleotide biosynthesis and not genes directly related to sugar metabolism. As a result, there currently is a surprising lack of riboswitches that sense any of the diverse (unmodified or phosphorylated) sugar molecules, including such universally important molecules such as glucose or fructose. This absence of RNA aptamers extends into the field of synthetic RNA aptamers that are produced by directed evolution methods. Perhaps RNA-based binding pockets for unmodified sugar molecules are difficult to create, given the possible challenge sugars present to receptors. Sugars such as glucose present only hydroxyl groups as molecular recognition contacts, which might appear like aqueous solvent to an aptamer – thus making selective recognition more difficult compared to many other riboswitch ligands. As with all other ligand categories, protein factors could have replaced sugar-responsive riboswitches via evolutionary competition.

### Riboswitches for Other Ligands are Rare.

Only a single, poorly defined riboswitch ligand resides outside of the categories discussed in detail above. The azaaromatic riboswitch class^116^ is somewhat enigmatic because the precise ligand sensed by these RNAs remains uncertain, although it is likely to be one or more members of a family of planar, hydrophobic compounds. As noted above, the PadR-type protein representatives that appear to regulate the same gene when the riboswitch is absent are known to serve as broad sensors of hydrophobic compounds that can be toxic^139,140^. Thus, it is likely that this riboswitch class senses compounds whose toxicity is overcome by the action of the YjdF protein (e.g. *B. subtilis* UniProt ID: O31647). Unfortunately, the substrate specificity and biochemical function of this protein (possibly a transporter) both remain unexplored.

Because almost no riboswitch ligands fall outside the major categories reflecting fundamental biochemical processes, existing riboswitches predominantly are used by bacteria to monitor ligands that reflect the status of major metabolic or physiological processes. The roles performed by these RNAs likewise could have been similar in ancient organisms who could only rely on RNA to form receptors and regulators until encoded protein synthesis emerged in evolution.

## CONCLUDING REMARKS

The analysis described herein is limited in two ways. First, the current list of riboswitch classes most likely represents only a small fraction of the total number that are present in extant bacterial species. Unfortunately, expanding this collection is constrained by the difficulty in finding rare classes by using computational or other search strategies. The discovery process is further restricted because the genomic sequences for all bacterial species on the planet cannot realistically be gathered. Second, the riboswitches present in today’s organisms are the products of both ancient and more recent evolutionary forces. Thus, it is not certain that the RNA aptamers of modern riboswitches represent the structures and functions used by organisms from the RNA World. However, the capabilities demonstrated by natural riboswitches provides a sampling of the capabilities that could have been exploited by ancient forms of life if they were restricted to using only RNA as a medium for forming molecular sensors and switches.

Although the current list of experimentally validated riboswitch classes (Figure 2) is undoubtedly incomplete, it almost certainly includes nearly all riboswitch classes that rank in the top 25 based on abundance. As additional classes are discovered, it is likely that many others already on the list will prove to be abundant relative to those discovered later. This is expected to be true because the bioinformatics methods used to discover novel candidates^143–146^ have a higher probability of encountering abundantly represented classes. From this incomplete collection it is striking to see that the ligands for these riboswitches are clustered near to important chemical and metabolic processes. Indeed, these RNAs alone would have provided primitive forms of life with mechanisms to manage a variety of essential pathways for a complex metabolic state like that observed in modern organisms.

There are, however, some glaring gaps in the demonstrated capabilities of natural riboswitches. Although phosphates are common molecular recognition determinants for many riboswitch aptamers, none of the ligands exhibit a predominant role in triggering the regulation of genes involved in phosphorus homeostasis. Similarly, although many riboswitches exploit ribose or deoxyribose moieties of ligands as major molecular recognition determinants, the regulation of sugar metabolism is almost entirely ignored. The only prominent exception is exhibited by the *glmS* ribozyme^141,142^, which senses the modified sugar GlcN6P to regulate its production.

Perhaps most notable is the absence of riboswitch ligands that are related to lipid or terpenoid metabolism. The charged nature of RNA polymers likely makes it difficult to form numerous different binding pockets for strongly hydrophobic molecules. This absence of hydrophobic ligands does not help overcome the general concern that RNA-based forms of life might have had a difficult challenge of managing metabolic pathways for lipid biosynthesis or even forming membrane associations. This problem could be partly overcome by covalently attaching RNAs to hydrophobic molecules such as fatty acids or terpenoid compounds. Unfortunately, another notably absent riboswitch class is for the ligand CoA, or any of its acylated derivatives. The adenosyl and pantothenate moieties of CoA should serve as excellent molecular recognition surfaces for riboswitch aptamers, where the hydrophobic acyl group would not need to provide contact points. Such aptamers could simply use steric constraints to measure the length of the acyl group to provide additional selective recognition capabilities.

Other obviously suitable ligands for extant riboswitch classes are also currently missing. In addition to CoA, other enzyme cofactors such as pyridoxal phosphate, CoQ, biotin, heme, and lipoic acid have yet to be found (Figure 3A). A notable theme emerges on examination of some of these gaps in riboswitch sensing. CoA, biotin, and lipoic acid are essential in modern metabolic pathways for lipid production and degradation. These processes have been proposed^16^ to have emerged later in evolution, after encoded protein synthesis had already begun. Furthermore, CoQ is specially adapted to localize to the hydrophobic portion of phospholipid bilayers of cell membranes^51^. If the fatty acid tails of phospholipids were not produced by RNA World organisms, perhaps there was no need for riboswitches that sensed CoQ or any of the other coenzymes used for fatty acid biosynthesis or degradation. Of course, numerous other explanations for missing riboswitch ligands are plausible, including ligand instability or reactivity with RNA. Therefore, the merits of each missing ligand candidate need to be evaluated based on its chemical properties, history in evolution, and requirements for monitoring, among many other factors.

The list of most-predictable riboswitch ligands extends to RNA-based signaling molecules such as cAMP, cGMP, and Ap_4_A (Figure 3B). Finally, there are almost no riboswitch ligands that can be grouped into the “other compounds” category. This might be partly due to the strong bias for ligands carrying nucleotide-derived chemical moieties. Regardless, there are no riboswitches for the many fundamental compounds involved in glycolysis or the citric acid cycle. The closest riboswitches come to affecting these common energy metabolism pathways is the glycine riboswitch^128^, which commonly regulates genes for the glycine cleavage system that directs carbon from glycine into the formation of acetyl-CoA that could then enter the citric acid cycle. Given how obvious these potential riboswitch ligands appear to be, it seems likely that at least some of these foreseen riboswitch classes might reside among the long list of ‘orphan’ riboswitch candidates (likely over 100) whose ligands remain to be verified^143–147^.

## Figures and Tables

**Figure 1.**
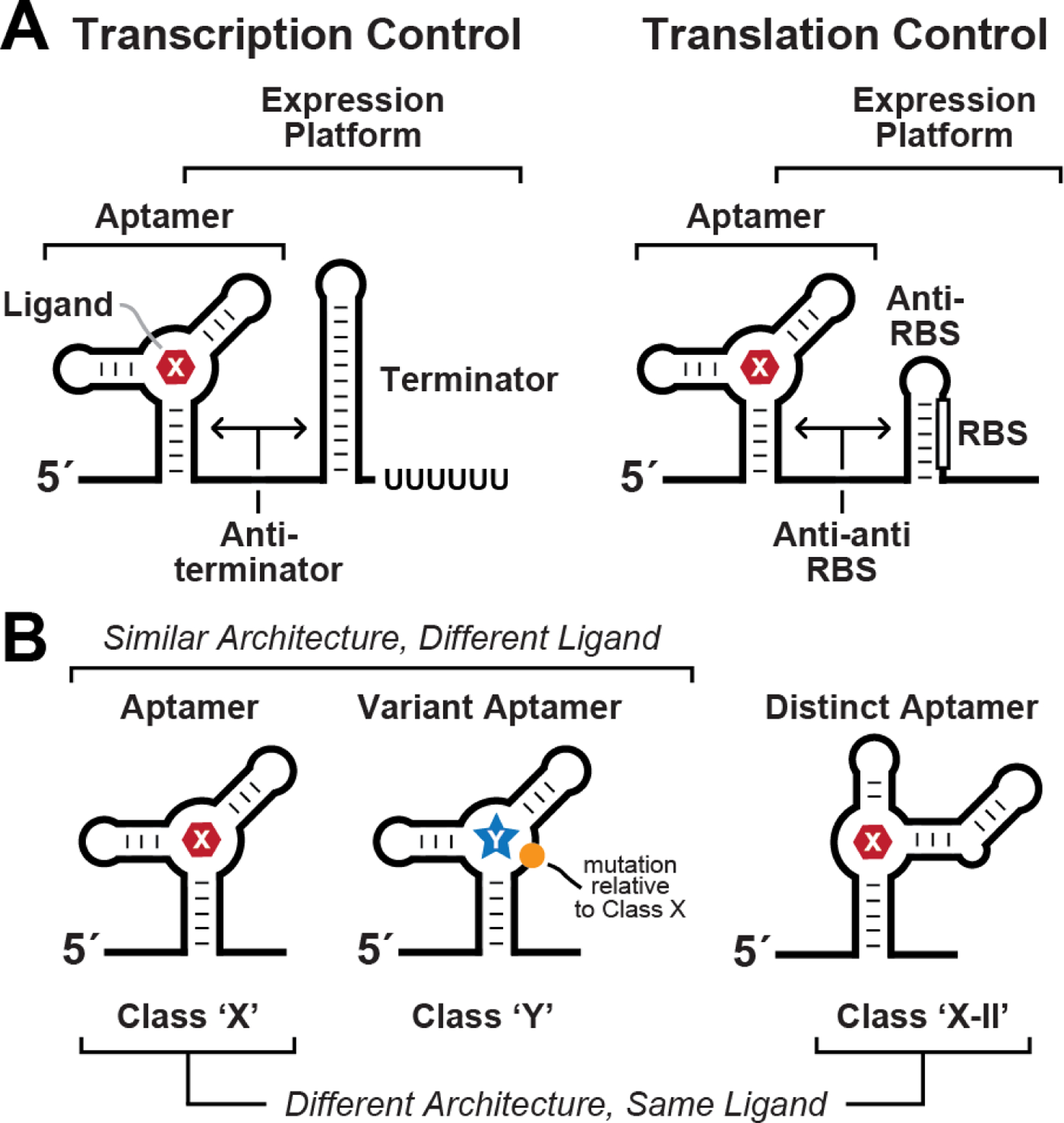
General features of bacterial riboswitches. (A) The predominant architectural features and gene control mechanisms of bacterial riboswitches. Most riboswitches carry a single ligand-binding aptamer domain that precedes and partially overlaps an expression platform structure capable of preventing or promoting gene expression. The two most common expression platform mechanisms exploit ligand-modulated hairpin substructures that terminate transcription (left) or that block ribosome access to the ribosome binding site (RBS) (right), although other mechanisms also exist^3–6^. (B) Riboswitches are classified and named based on distinct ligand binding specificities and distinct aptamer structures. The “-II” notation identifies the second class for a given ligand.

## ABBREVIATIONS

2′-dG: 2′-deoxyguanosine
10f-THF: 10-formyl-tetrahydrofolate
ADP: adenosine 5′-diphosphate
AdoCbl: adenosylcobalamin
Ap_4_A: diadenosine tetraphosphate
AqCbl: aquocobalamin
cAMP: 3′,5′-cyclic adenosine monophosphate
c-di-AMP: cyclic di-adenosine monophosphate
c-di-AMP-GMP: cyclic di-guanosine monophosphate
c-di-GMP: cyclic adenosine monophosphate-guanosine monophosphate
FMN: flavin mononucleotide
GlcN6P: glucosamine-6-phosphate
HET-P: 4-methyl-5-β-hydroxyethylthiazole phosphate
HMP-PP: 4-amino-2-methyl-5-hydroxymethylpyrimidine pyrophosphate
MoCo: molybdenum cofactor
NAD^+^: nicotinamide adenine dinucleotide
ORF: open reading frame
ppGpp: guanosine tetraphosphate
PRA: phosphoribosylamine
preQ_1_: prequeuosine-1
PRPP: phosphoribosyl pyrophosphate
RBS: ribosome binding site
SAH: *S*-adenosylhomocysteine
SAM: *S*-adenosylmethionine
THF: tetrahydrofolate
TPP: thiamin pyrophosphate
uORF: upstream open reading frame
WCo: tungsten cofactor
ZTP: 5-amino 4-imidazole carboxamide riboside 5′-triphosphate
